# Trends in Indicators of Violence Among Adolescents in Europe and North America 1994–2022

**DOI:** 10.3389/ijph.2025.1607654

**Published:** 2025-02-24

**Authors:** Michal Molcho, Sophie D. Walsh, Nathan King, William Pickett, Peter D. Donnelly, Alina Cosma, Frank J. Elgar, Kwok Ng, Lilly Augustine, Marta Malinowska-Cieślik, Ylva Bjereld, Wendy Craig

**Affiliations:** ^1^ School of Education, University of Galway, Galway, Ireland; ^2^ Department of Criminology, Bar-Ilan University, Ramat Gan, Israel; ^3^ Department of Public Health Sciences, Queen’s University, Kingston, ON, Canada; ^4^ Department of Health Sciences, Brock University, St. Catharines, ON, Canada; ^5^ Medical School, University of St Andrews, St Andrews, Scotland, United Kingdom; ^6^ Dalla Lana School of Public Health, University of Toronto, Toronto, ON, Canada; ^7^ Trinity Centre for Global Health, School of Psychology, Trinity College Dublin, Dublin, Ireland; ^8^ Olomouc University Social Health Institute, Palacky University Olomouc, Olomouc, Czechia; ^9^ Department of Psychology, McGill University, Montreal, QC, Canada; ^10^ Faculty of Education, University of Turku, Turku, Finland; ^11^ Department of Physical Education and Sport Sciences, Physical Activity for Health Research Centre, University of Limerick, Limerick, Ireland; ^12^ Institute of Innovation and Sports Science, Lithuanian Sports University, Kaunas, Lithuania; ^13^ Faculty of Sport and Health Sciences, University of Jyväskylä, Jyväskylä, Finland; ^14^ CHILD, School of Learning and Communication, Jönköping University, Jönköping, Sweden; ^15^ Department of Environmental Health, Faculty of Health Sciences, Jagiellonian University Medical College, Krakow, Poland; ^16^ Department of Social Work, University of Gothenburg, Göteborg, Sweden; ^17^ Department of Psychology, Queen’s University, Kingston, ON, Canada

**Keywords:** adolescent health, violence, bullying, fighting, cyberbullying

## Abstract

**Objectives:**

To describe age and gender specific time trends in adolescent violence across 19 countries over 28 years.

**Methods:**

The paper presents analysis of eight cycles of the Health Behaviour in School-aged Children (HBSC) Study from 1994–2022, involving 789,531 children aged 11, 13, and 15. Indicators of violence included physical fighting, school bullying and cyberbullying (from 2018). Log-binomial regression models were used to test for linear temporal trends, with Generalized Estimating Equations used to account for clustering by country.

**Results:**

School bullying perpetration and victimization declined over time in each age/gender group in most countries. Similar declines were reported for frequent physical fighting among boys (all ages) and girls (age 15 only). The prevalence of violent behaviour was almost universally higher in boys in the early cycles than in girls, but this gender difference attenuated over time. For cyberbullying, significant increases were observed since 2018 in all groups except age 15 girls in most countries.

**Conclusion:**

This analysis of a large cross-national dataset suggests a decline in traditional forms of adolescent violence. However, the increases in cyberbullying warrant further monitoring.

## Introduction

Peer violence among young people, including bullying and physical fighting, is a major public health concern with short-term and chronic health consequences. The impacts of violence and aggression on adolescent health and wellbeing are well documented [1, 2]. During adolescence, engagement in physical fights is associated with increased risk for injury [3, 4], substance use [5, 6], other problem behaviours [7, 8], and lower life satisfaction [9, 10]. Bullying others in school is associated with an increased risk of a range of physical, emotional, and behavioural problems [11–13]. Victims of school bullying are more likely to suffer from mental health problems and low self-esteem [2, 14–18], and report poor academic performance [13, 19, 20]. Even exposure to bullying as a bystander can harm adolescent health and wellbeing [21]. In the long-term, adolescent violence correlates with adult trajectories that include impaired employment and education, as well as criminal behaviour [22, 23].

The origins and nature of aggressive behaviour are complex, and engagement in violence results from the intersection of personal, social, community, societal, and environmental characteristics. From a theoretical perspective, ecological theories of violence [24, 25] describe adolescent involvement in violence as a result of multiple proximal variables (e.g., individual characteristics [26] and values [27]) and distal norms [28], and cultural expectations [29]. These ecological theories suggest that the prevalence and expression of adolescent violence may change over time, alongside societal changes (e.g., norms, technology) which can relate to violent behaviour. Other determinants of adolescent violent behaviour include relationships with family [30], exposure to violent media in childhood [31], and school climate [32].

Despite what is known about individual-level determinants of adolescent violence, large-scale studies that document population level changes over time are rare. Past research exploring trend data up to 2010 reported a decrease in the prevalence of a number of aggressive behaviours, including physical fighting [33] and bullying perpetration [34] and victimization [35, 36], which have been attributed to societal changes. Since the early 1990s, there has been an increase in published work in the field of bullying [37], which has coincided with the increased awareness of indicators of violence such as bullying, and its associated negative consequences. This interest in international trends has been fostered by the introduction of bullying prevention programs, mostly in schools [38, 39]. These prevention efforts may explain decreases in reports of bullying observed regionally [40], although very few countries have conducted randomized controlled trials and other high-level evaluations on these interventions. Interestingly, despite the many known effects of engagement in physical fighting during adolescence, this behaviour has not received the same level of attention in either research or in prevention and intervention programming as bullying.

Adding to the complexity of this issue, with the rise in popularity of social media platforms, cyberbullying has emerged as a new expression of adolescent violence. While there is no agreed upon definition of cyberbullying [41], and it is considered as any form of bullying being acted out through electronic means [42]. Cyberbullying presents a notable departure from conventional bullying due to the potential anonymity of perpetrators and the capacity for occurrences at any hour [43]. Cyberbullying is both prevalent and associated with increased social media use [12], and has known harms to emotional and mental health [41, 44, 45]. Although it is not clear whether cyberbullying is new phenomena replacing traditional bullying or is simply an extension of traditional face-to-face bullying, often the same people who perpetrate traditional face-to-face bullying or experience related victimization are also engaged in cyberbullying [41, 46]. Features of cyberbullying such as lack of adult supervision online, lack of awareness, lack of interpersonal contact and direct feedback from the victim, and its broad reach, make it particularly difficult to protect and support adolescents who are either perpetrators, victims, or bystanders of cyberbullying.

Health Behaviour in School-aged Children (HBSC) is a cross-national adolescent health study that has collected data for over 40 years. HBSC first collected data on school bullying in 1994, long before this was a popular area of scientific discourse [36]. In 2002, the HBSC study included an indicator on physical fighting, which until then, was used only in a small number of countries. Since then, data on both bullying and physical fighting have been collected in every survey cycle. In 2018, the international survey protocol introduced mandatory questions on cyberbullying, providing an opportunity to present recent changes in cyberbullying, and to examine how changes in cyberbullying align with the overall time trends in violent behaviours in adolescence. This 28-year history makes HBSC unique as a cross-national, population health study that now collects five standard indicators of adolescent violence, permitting analysis of trends in the engagement of adolescents in violent behaviours, both overall and within specific groups defined by age and gender.

The current analysis explores, over a 28-year period, trends in the occurrence of adolescent violence in the 19 countries that have used the available indicators since their introduction to HBSC. Its specific objectives were to: (1) describe changes in the prevalence of these five indicators (frequent physical fighting, school bullying perpetration and victimization, cyberbullying perpetration and victimization) across countries and over time; then (2) identify overall and age/gender-specific trends in violence within these countries. This included exploring the prevalence of cyberbullying before and in the late stages of, or after (depending on the timing of data collection in each country), the COVID-19 pandemic, and their alignment with changes over time in other aggressive behaviours included in this study.

## Methods

### Study Population and Procedures

Initiated in 1982, the HBSC study collects data from nationally representative samples of 11, 13, and 15-year-old school children every 4 years in each of the participating countries (44 countries in 2022). Data were collected in classrooms using an anonymous self-administered questionnaire and followed the common HBSC research protocol for both sampling and data collection [46]. Classes within schools formed the sampling units, with variations in sampling criteria suited to country-level circumstances. Countries are required to sample children from schools representing at least 95% of this target population in their national sampling frames, and where countries oversampled subpopulations (e.g., by geography and ethnicity), and, where deemed appropriate, standardized weights were created to ensure representativeness.

The current analysis presents data from the 19 countries and regions that collected data from eight survey cycles, from 1993/1994 to 2021/22. These 19 countries are Austria, Belgium (Vlams Gewest region), Belgium (Wallonia region), Canada, Czech Republic, Denmark, Estonia, Finland, France, Germany, Greenland, Hungary, Israel, Latvia, Lithuania, Poland, Scotland, Sweden, and Wales. Each participating country obtained approval to survey each of the cycles from the ethics review board or equivalent regulatory body associated with the institution conducting each respective national survey. Participation was voluntary, and consent (explicit or implicit) was sought from school administrators, parents, and children as per national human subject requirements.

### Indicators of Adolescent Violence

#### Bullying (2 Indicators)

Questions on school bullying, developed by Olweus [47], were introduced to the HBSC study in the 1994 cycle, and questions on cyberbullying were introduced to the study in 2018, based on the Olweus scale [48] and modified to the cyber context.

##### Perpetration

In the 1994 and 1998 surveys, the question for bullying was phrased “How often have you taken part in bullying other students in school this term?” with response options “I haven’t bullied others in school this term,” “once or twice,” “sometimes,” “about once a week,” “several times a week.” From 2002 onwards, a slightly different phrasing was used: “How often have you taken part in bullying other students at school in the past couple of months?” with response options “I haven’t bullied other students in the past couple of months,” “it has only happened once or twice,” “two or three times a month,” “about once a week,” “several times a week.” From 2018 onwards, the question was changed to “How often have you taken part in bullying other persons at school in the past couple of months?” A cut-off of “2–3 times a month or more” (or “sometimes” pre-2002) was used to capture a regular pattern of perpetration. This cut-off that was originally proposed by Solberg and Olweus [49], has been consistently used in HBSC publications and reports [35, 45].

##### Victimization

In the 1994 and 1998 surveys, the question for bullying victimization was phrased “How often have you been bullied at school this term?,” and from 2002 onwards, a slightly different phrasing was used: “How often have you been bullied at school at school in the past couple of months?.” The respective response categories were analogous to those for bullying perpetration, with the same changes and cut-off for bullying perpetration as presented above.

#### Cyberbullying (2 Indicators)

##### Perpetration

Since 2018, participants were asked “In the past couple of months, how often have you taken part in cyberbullying (e.g., sent mean instant messages, email or text messages; wall postings; created a website making fun of someone; posted unflattering or inappropriate pictures online without permission or shared them with others)?.” Response options were the same as for school bullying. A cut-off of “at least once or twice in the past couple of months” was used to capture engagement in cyberbullying.

##### Victimization

The question on cyberbullying victimization and the respective response categories were analogous to those for cyberbullying perpetration, using the same cut-off.

We used a different cut-off for cyberbullying compared to school bullying, as online posts can be seen multiple times, representing repeated bullying rather than a single face-to-face incident [43].

#### Frequent Physical Fighting

Participants were asked “During the past 12 months, how many times were you in a physical fight?” with answering categories “I have not been in a physical fight during the past 12 months,” “1 time,” “2 times,” “3 times,” “4 times or more.” A cut-off of 3 times or more was used to capture recurrent involvement in fighting [33].

#### Stratification Variables

Other variables used in the analyses were gender (boy or girl), age group (11, 13, and 15 years), and country or region.

### Statistical Analysis

All analyses were performed in SAS 9.4 (SAS Institute, Cary NC) and weighted using country-specific weights, where such were available, to ensure national representativeness. Using the pooled overall samples from the 19 countries, we first estimated the prevalence of each of the five indicators of adolescent violence, as available, and stratified by age group, gender, and survey cycle. 95% confidence intervals (CIs) were estimated with adjustment for clustering by country/region. Next, log-binomial regression models that included HBSC cycle (year) as an independent continuous variable and each indicator of adolescent violence as the dependent variable were used to test for linear trends over time (bullying and fighting) [50], or changes between 2018 and 2022 cycles (cyberbullying only). Models accounted for clustering by country using Generalized Estimating Equations (GEEs) [51]. Interactions terms were used to test whether the linear trends differed by gender. Significance level in all these analyses was set at p < 0.05. At the country level, we estimated the absolute change in the prevalence of each indicator (and associated confidence interval [CI], adjusted for clustering by school) within each age and gender strata from the first to last cycle that the indicator was available. Based upon whether or not the CI included 0, and the direction of effects, the number of countries that demonstrated a decrease, increase, or no change in prevalence within the strata were identified.

## Results

Schoolchildren (N = 789,531) in 19 countries and regions provided complete answers on age, gender, and at least one of the bullying or fighting variables over the eight survey cycles from 1994 to 2022. Table 1 describes the sample size by country, gender, and age group.

**TABLE 1:** Description of international Health Behaviour in School-aged Children (HBSC) study samples, by survey cycle in 19 countries (1994-2022).

	1994				1998				2002				2006				2010				2014				2018				2022			
	No.				No.				No.				No.				No.				No.				No.				No.			
Total participants	81,094				79,355				89,780				93,429				100,716				100,555				110,666				133,936			
By country																																
Minimum	1,243				1,559				844				1,324				1,165				966				1,156				1,089			
Median	4,118				4,499				4,388				4,748				4,779				5,220				4,395				5,155			
Maximum	6,722				6,481				8,175				7,178				15,561				12,753				14,354				31,866			
By gender and age		No.		%		No.		%		No.		%		No.		%		No.		%		No.		%		No.		%		No.		%	
Boys																																
11 years		13,448		34.2		13,563		35.0		15,351		35.1		14,614		32.1		16,248		33.0		15,836		31.8		18,226		33.6		21,087		31.9	
13 years		13,154		33.5		13,196		34.1		14,905		34.1		15,461		33.9		16,627		33.8		17,149		34.5		18,891		34.9		23,121		35.0	
15 years		12,686		32.3		11,976		30.9		13,474		30.8		15,505		34.0		16,365		33.2		16,791		33.7		17,082		31.5		21,844		33.1	
Girls																																
11 years		14,285		34.2		13,898		34.2		15,652		34.0		15,240		31.9		16,861		32.8		16,464		32.4		18,678		33.1		21,596		31.8	
13 years		14,071		33.7		13,747		33.8		15,839		34.4		16,145		33.7		17,393		33.8		17,093		33.7		19,409		34.4		23,183		34.2	
15 years		13,451		32.2		12,974		31.9		14,559		31.6		16,464		34.4		17,222		33.5		17,222		33.9		18,380		32.6		23,106		34.0	

**FIGURE 1:**
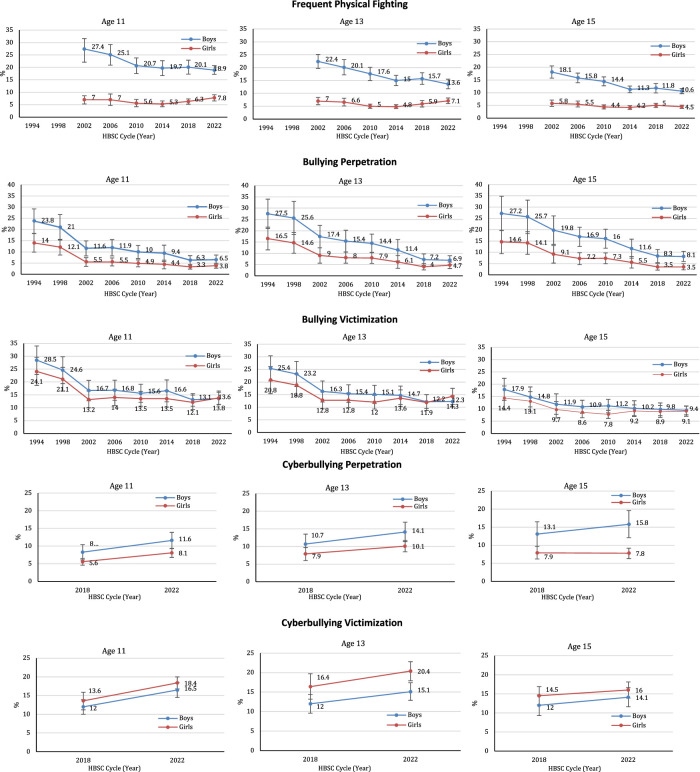
Prevalence of five indicators of violence in Boys and Girls in 19 Countries, by Health Behaviour in School-aged Children (HBSC) Cycle and age group (Health Behaviour in School-aged Children study, 1994–2022 for 19 countries).

The prevalence of each of the five indicators of violence is described in Table 2, and further illustrated in the series of graphs presented in Figure 1. Among boys in the overall sample, the prevalence of frequent physical fighting (11 years old: 27.4% in 2002 and 18.9% in 2022, p < 0.001; 13 years old: 22.4% in 2002 and 13.6% in 2022, p < 0.001; 15 years old: 18.1% in 2002 and 10.6% in 2022, p < 0.001), bullying perpetration (11 years old: 23.8% in 1994 and 6.5% in 2022, p < 0.001; 13 years old: 27.5% in 1994 and 6.9% in 2022, p < 0.001; 15 years old: 27.2% in 1994 and 8.1% in 2022, p < 0.001), and bullying victimization (11 years old: 28.5% in 1994 and 13.6% in 2022, p < 0.001; 13 years old: 25.4% in 1994 and 12.3% in 2022, p < 0.001; 15 years old: 17.9% in 1994 and 9.4% in 2022, p < 0.001), decreased over time. Among girls, similar decreases were observed in each age group for bullying perpetration (11 years old: 14.0% in 1994 and 3.8% in 2022, p < 0.001; 13 years old: 16.5% in 1994 and 4.7% in 2022, p < 0.001; 15 years old: 14.6% in 1994 and 3.5% in 2022, p < 0.001) and victimization (11 years old: 24.1% in 1994 and 13.8% in 2022, p < 0.001; 13 years old: 20.8% in 1994 and 14.3% in 2022, p < 0.001; 15 years old: 14.4% in 1994 and 9.1% in 2022, p < 0.001), but not for physical fighting. The prevalence of each of these three indicators were initially much higher for boys in comparison to girls, but this gender gap became smaller in more recent survey cycles. As a result, the linear temporal decline in bullying victimization was greater in boys than girls across all age groups (p-interaction <0.05). This finding also appeared to be true for bullying perpetration, but only for the 15-year-old age group (p-interaction = 0.06). Physical fighting was most prevalent among 11-year-old boys, bullying others was more prevalent among older adolescents, and bullying victimization was more prevalent among younger adolescents (both boys and girls). Overall, a sharp decline in bullying (both perpetration and victimization) was evident between 1994 and 2002, with a steadier decline since then, whereas the prevalence of frequent physical fighting declined more steadily over time. An examination of the interaction between gender and HBSC survey cycle found that the linear trends for physical fighting were significantly different for boys and girls in all three age groups (p < 0.001). Frequent physical fighting significantly decreased in boys from 2002 to 2022 but remained relatively low and stable in girls across the same time period (see Figure 1).

**TABLE 2:** Linear time trends and prevalence of five indicators of adolescent violence (frequent physical fighting; bullying perpetration then victimization; cyberbullying perpetration and victimization) in 19 countries across the 1994 and 2022 Health Behaviour in School-aged Children (HBSC) survey cycles, by sex and age group.

	1994		1998		2002		2006		2010		2014		2018		2022		p-trend*
	%	95% CI	%	95% CI	%	95% CI	%	95% CI	%	95% CI	%	95% CI	%	95% CI	%	95% CI	
Frequent Physical Fighting																	
Boys																	
11 years					27.4	22.1–31.6	25.1	20.9–29.2	20.7	17.5–23.8	19.7	16.7–22.7	20.1	17.3–22.9	18.9	17.2–20.7	<0.001
13 years					22.4	19.7–25.1	20.1	17.2–23.1	17.6	15.0–20.1	15.0	13.0–17.0	15.7	13.5–18.0	13.6	11.8–15.5	<0.001
15 years					18.1	15.7–20.5	15.8	13.9–17.7	14.4	12.7–16.2	11.3	10.0–12.6	11.8	9.9–13.6	10.6	9.6–11.6	<0.001
Girls																	
11 years					7.0	5.3–8.6	7.0	4.7–9.3	5.6	4.2–7.1	5.3	4.2–6.5	6.3	5.2–7.3	7.8	6.5–9.0	0.83
13 years					7.0	5.6–8.4	6.6	5.1–8.1	5.0	4.2–5.8	4.8	4.1–5.6	5.9	4.7–7.2	7.1	6.0–8.2	0.81
15 years					5.8	4.6–7.1	5.5	4.4–6.7	4.4	3.6–5.2	4.2	3.4–4.9	5.0	4.2–5.8	4.5	3.8–5.1	0.003
Bullying Perpetration																	
Boys																	
11 years	23.8	18.3–29.2	21.0	15.3–26.7	11.6	8.2–14.9	11.9	8.2–15.5	10.0	7.0–12.9	9.4	5.7–13.0	6.3	4.1–8.3	6.5	4.3–8.6	<0.001
13 years	27.5	21.0–34.0	25.6	18.3–33.0	17.4	12.4–22.4	15.4	10.6–20.2	14.4	10.3–18.5	11.4	6.6–16.1	7.2	4.7–9.7	6.9	4.9–8.9	<0.001
15 years	27.2	19.6–34.8	25.7	18.3–33.1	19.8	13.5–26.1	16.9	12.6–21.1	16.0	11.8–20.2	11.6	7.4–15.8	8.3	5.4–11.2	8.1	5.9–10.4	<0.001
Girls																	
11 years	14.0	9.9–18.2	12.1	8.6–15.6	5.5	3.5–7.4	5.5	3.7–7.3	4.9	3.3–6.4	4.4	2.4–6.3	3.3	2.3–4.3	3.8	2.7–4.9	<0.001
13 years	16.5	11.5–21.6	14.6	10.0–19.2	9.0	5.6–12.4	8.0	5.6–10.5	7.9	5.5–10.4	6.1	3.3–9.0	4.0	2.6–5.5	4.7	3.2–6.1	<0.001
15 years	14.6	9.4–19.7	14.1	9.1–19.1	9.1	5.2–13.0	7.2	4.5–9.8	7.3	4.9–9.7	5.5	3.0–8.1	3.5	2.1–4.9	3.5	2.2–4.9	<0.001
Bullying Victimization																	
Boys																	
11 years	28.5	22.9–34.0	24.6	19.4–29.8	16.7	12.7–20.6	16.8	12.9–20.7	15.6	12.1–19.1	16.6	12.3–20.8	13.1	10.6–15.5	13.6	11.3–16.0	<0.001
13 years	25.4	20.4–30.4	23.2	18.2–28.2	16.3	12.1–20.4	15.4	11.9–18.9	15.1	11.5–18.7	14.7	11.1–18.4	12.2	9.5–15.0	12.3	10.2–14.5	<0.001
15 years	17.9	13.5–22.4	14.8	10.8–18.9	11.9	7.8–16.1	10.9	8.2–13.5	11.2	8.4–13.9	10.2	7.2–13.3	9.8	7.2–12.4	9.4	7.6–11.2	0.001
Girls																	
11 years	24.1	18.7–29.6	21.1	16.5–25.7	13.2	10.0–16.5	14.0	10.8–17.2	13.5	10.6–16.4	13.5	10.3–16.7	12.1	9.3–14.9	13.8	11.2–16.5	0.0001
13 years	20.8	15.4–26.1	18.8	14.5–23.1	12.8	9.4–16.1	12.8	9.9–15.7	12.0	9.3–14.7	13.6	10.3–16.9	11.9	8.7–15.0	14.3	11.1–17.5	0.02
15 years	14.4	9.6–19.3	13.1	9.1–17.0	9.7	6.3–13.0	8.6	6.4–10.8	7.8	6.1–9.4	9.2	6.9–11.6	8.9	6.5–11.4	9.1	7.0–11.1	0.03

Cyberbullying perpetration **													2018		2022		p-trend*
													%	95% CI	%	95% CI	
Boys																	
11 years													8.3	6.1–10.4	11.6	9.3–13.9	<0.001
13 years													10.7	7.9–13.5	14.1	11.4–16.9	<0.001
15 years													13.1	9.7–16.5	15.8	12.1–19.4	0.04
Girls																	
11 years													5.6	4.6–6.5	8.1	6.8–9.4	<0.001
13 years													7.9	6.0–9.7	10.1	8.5–11.7	0.003
15 years													7.9	6.2–9.7	7.8	6.3–9.2	0.54
Cyberbullying Victimization **																	
Boys																	
11 years													12.0	10.0–13.9	16.5	14.5–18.5	<0.001
13 years													12.0	9.6–14.4	15.1	12.9–17.4	<0.001
15 years													12.0	9.3–14.7	14.1	11.6–16.6	0.01
Girls																	
11 years													13.6	11.2–15.9	18.4	16.8–20.0	<0.001
13 years													16.4	13.2–19.7	20.4	17.9–22.8	0.003
15 years													14.5	12.1–16.9	16.0	13.9–18.1	0.19

Notes: (1) 95% confidence intervals are adjusted for clustering by country, (2) * p-value for linear trend across years (HBSC survey cycles), (3) ** cyberbullying items not available in Greenland, both 2018 and 2022 cycles.

Table 2; Figure 1 additionally provide estimates of the prevalence of cyberbullying in the two available cycles (2018, 2022; with 2022 conducted at the tail end of the COVID-19 pandemic). Except for perpetration among 15-year-old girls (p-interaction <0.01), the prevalence of cyberbullying perpetration and victimization was higher in 2022 compared to 2018 in boys and girls in all three age groups. A gendered pattern was evident, with boys in all age groups reporting a higher prevalence of cyberbullying perpetration; and girls in all age groups reporting a higher prevalence of victimization, suggesting potential vulnerability.

Based upon changes observed between the first and last available cycle of data for each indicator, Table 3 identifies the number of countries for which increases, decreases or no change was observed for the five indicators of adolescent violence, stratified by age group and gender (note: these changes are summarized in Supplementary Figures S2, S3, by country). For frequent physical fighting, among boys, decreases were reported in most countries. Among girls, the prevalence of fighting has remained stable. Bullying perpetration decreased in most countries for both genders and all age groups. The pattern for bullying victimization was mixed. In most countries, the prevalence of bullying victimization decreased for boys across all age groups, with no change in a small number of countries. Among girls, the prevalence of bullying victimization decreased in most countries for 11-year-olds; however, this pattern became less pronounced in the older age groups. For the available indicators of cyberbullying, the prevalence of perpetration and victimisation was generally the same or higher in 2022 compared to 2018, reflective of the overall increase in these behaviours in most countries.

**TABLE 3:** Number of countries (n = 19) that had a decrease, no change, or increase* in the prevalence of five indicators of adolescent violence, from the first to last cycle the indicator was available (Health Behaviour in School-aged Children study, 1994–2022 for 19 countries).

Frequent physical Fighting (2002–2022)		Decreased	No change	Increased
Boys	11 years	13	5	1
	13 years	13	6	0
	15 years	15	4	0
Girls	11 years	2	14	3
	13 years	3	10	6
	15 years	5	12	2
Bullying Perpetration (1994 to 2022)				
Boys	11 years	17	2	0
	13 years	16	3	0
	15 years	14	5	0
Girls	11 years	15	4	0
	13 years	16	3	0
	15 years	12	7	0
Bullying Victimization (1994 to 2022)				
Boys	11 years	16	2	1
	13 years	14	5	0
	15 years	11	6	2
Girls	11 years	13	4	2
	13 years	11	5	3
	15 years	9	7	3
Cyberbullying Perpetration (2018 to 2022)**				
Boys	11 years	0	8	10
	13 years	0	8	10
	15 years	1	10	7
Girls	11 years	0	8	10
	13 years	0	13	5
	15 years	5	10	3
Cyberbullying Victimization (2018 to 2022)**				
Boys	11 years	0	8	10
	13 years	0	7	11
	15 years	1	10	7
Girls	11 years	0	8	10
	13 years	0	11	7
	15 years	0	14	4

Note: *Changes (decrease, no change, or increase) based on whether 95% CI interval for absolute change overlapped 0, (2) **18 of 19 countries (cyberbullying items not available in Greenland).

## Discussion

This cross-national analysis examined temporal trends in the prevalence of five indicators of adolescent violence in 19 countries and regions in Europe, the Middle East and Canada. Using representative cross-national samples compiled via a standardized survey protocol over 28 years, we explored the potential similarity of time trends in adolescent violence within countries, age groups and by gender. To our knowledge, this is the first study to compile such an in-depth exploration of trends in adolescent involvement in several indicators of peer violence over an almost three-decade time span. The results are both unique to the public health literature and of significant value for policy development. There were three major findings. *First,* while there were some exceptions, we identified general declines in the prevalence of traditional indicators of school bullying (both perpetration and victimization) and physical fighting in the last 30 years. *Second,* while we observed strong gendered patterns especially in the early years of study, gender differences in the prevalence of bullying (especially bullying perpetration) and physical fighting became less pronounced over time. *Third*, in the last two cycles of data collection that spanned the COVID-19 pandemic, cyberbullying (both perpetration and victimization) increased in most countries, genders and age groups.

Our findings add to a body of literature that has demonstrated that engagement in face-to-face bullying and other aggressive behaviours has declined over time [33–35, 45]. Our data indicate that these declines were stronger in the 1990s and early 2000s and have slowed since. Increased social awareness and anti-bullying campaigns and programs in school can possibly explain some of the decline in bullying behaviour. Some have proposed that there is a floor effect, such that changes in social norms and culturally endorsed behaviours continue to contribute to adolescent violence becoming increasingly unacceptable [52], leaving the impact of personal and proximal factors as the major factors influencing aggressive behaviour, thus echoing the explanations suggested by various ecological models [25, 26]. As aggressive behaviours are becoming less socially condoned, individual traits that include self-regulation, self-esteem, empathy, life skills, and interpersonal competencies, as well as family and peer relationships and supports may be increasingly important as etiological factors [53]. Finally, it is important to recognize that violence and aggression share commonalities with other social behaviours in adolescents, which have demonstrated marked declines over the same period as our study. Many types of adolescent risk behaviour including alcohol use, smoking, and cannabis use exhibit similar temporal patterns [54–57], suggesting that decreases in aggressive behaviours may be considered as part of an overarching change in the social contexts and behaviours that are typical in young people’s lives. These trends are confounded by the increased engagement of young people in virtual behaviours including social media use, which too have been found to impact on young people’s mental health and wellbeing [43, 58].

Gendered patterns observed in the current analysis warrant comment. Consistent with past research, the prevalence of aggressive behaviour is higher in boys compared to girls [10, 33, 59]. This pattern, however, may be more nuanced when considering changes over time. While there was no significant interaction between gender and survey cycle for school bullying across age groups, it is important to note that in recent years, the past gender differences disappeared in the overall pooled analysis. This gender convergence has been observed in other adolescent male-typical behaviour such as alcohol drinking or smoking [60, 61]. Even more intriguing is the gender pattern in frequent physical fighting, where there was a significant difference in the decline among boys and girls, such that declines were only evident in boys, while for girls the prevalence remained stable or even increased over time. Some of the cross-national variations in these gender gaps may be attributed to structural gender inequalities. For example, countries with greater levels of gender inequality show the largest gaps in bullying at school and cyberbullying victmization [61]. Hence, it might be that the increasing levels of structural gender equality observed in the participating countries, with coincident decreases in the sanctioning of boys’ aggressive behaviours, have contributed to a narrowing of this historical gap between boys and girls. Others have suggested that gender differences in violence are physiological [62], and while the evidence on the association between testosterone levels and aggressive behaviour is inconsistent [36, 63], it is worth noting that the decline in fighting in boys reported here is consistent with reports on declines in testosterone levels [64]. It is, therefore, possible that this decline in testosterone levels contributes to this pattern in adolescent violence. From a public health perspective, understanding this gender trend may require more focus.

While based upon only two cycles of available data, the differences in cyberbullying observed between 2018 and 2022, consistent with similar increases observed over the last decade [65], are also notable. Such increases are concerning. As many as one in six participants experienced cyberbullying in the 2022 cycle, which is concerning given its potential effects on health, security, and wellbeing. While the origins of these effects remain speculative, many possible explanations will only emerge with time and further scrutiny. The increases may be attributable in part to public health measures imposed in response to the COVID-19 pandemic which lessened opportunity for in-person encounters, amplified time spent online and thus increased opportunity for virtual expressions of aggression. It is also possible that cyberbullying is replacing traditional forms of face-to-face bullying in some adolescents, suggesting that the problem of violence has not changed but is merely shifting online – although measuring cyberbullying in only two time points does not allow to fully assess this assumption. And finally, because the cyberbullying measures were asked in a slightly different manner than were the traditional face-to-face measures, perhaps there are methodological reasons for these differential increases. Only time and further study, both quantitative and qualitative, will increase understanding of these changes over time. Nonetheless, the observed increases in cyberbullying call for targeted gender-sensitive solutions that promote digital safety, empathy, and inclusive school cultures, irrespective of why these patterns have emerged [66].

Our study has many strengths. HBSC’s use of standard survey protocols and indicators of violence, which are among the very few that are comparable over this time frame, enabled the comparison of trends in adolescent violence across time, country, gender and age groups, which makes this analysis unique to the international adolescent violence literature. This analysis covered eight cycles of data collection conducted over 28 years, and involved reports from nearly 800,000 young people in 19 countries, making it one of the largest analyses of its kind. Our inclusion of five indicators of adolescent violence considered simultaneously is also unique, as it maps a broader change in violent behaviours, as opposed to previous literature, that predominantly focused on single indicators. Despite these strengths, there are limitations. Study limitations include our choice to focus the analysis on the 19 (of 44) countries in HBSC with complete data on available violence indicators over the 28 years, to enhance comparability in the trends analysis. These countries may not fully represent the broader experiences of young people across the globe. While this paper is not focused on country-level comparisons, we did find large country variations in the absolute change in the prevalence of violent behaviours, which could be explained by country differences in the prevalence of these behaviours at the base line [34, 35], however, including the baseline levels for each country is beyond the scope of this analysis. We also limited the analysis to the mandatory HBSC items describing peer violence and aggression and did not integrate items that were not used in all countries (for examples, items on types of bullying), and did nor measure exposure to violent media. More generally, all HBSC data are self-reported, and under-reporting of sensitive behaviours and hence prevalence levels is to be expected. How these sensitivities contribute to the temporal patterns remains unknown. Finally, challenges with translation from English to other native languages may have occurred, as well as the small changes in the response categories over time, leading to subtle interpretive differences that are hard to identify and describe, with certainty.

While mainly descriptive, our analysis provides foundational information which has the potential to guide national and international policy efforts. First, it provides new evidence supporting the identification of adolescent violence as a priority that crosses borders and geography, consistent with the positions of multiple bodies including UNICEF, the WHO, the OECD, and their member countries. Temporal patterns provide insights into how violence is playing out in the lives of young people, the role of gender in its expression, and how it is evolving in the face of a world where socialization is increasingly virtual. This research sets the stage for more in-depth etiological work, that focus on its key findings, and especially the evolving importance of cyberbullying as a public health and clinical problem and an understanding that adolescent violence should no longer be considered a predominantly male behaviour. Ongoing surveillance of these indicators can help to inform evaluative efforts conducted at the national and international level. The findings also make contributions to our understanding of these phenomena, providing information for the refinement of applied theory and practice. And finally, it demonstrates the importance of societal investment in cross-national studies that use common indicators and study approaches, to inform both theory and practice with evidence that is collected consistently, enhancing its comparability. HBSC is one of the few studies of its type that has done this for more than 40 years, and there is value in its sustained efforts.
