# Supplementary material for: Trends in Indicators of Violence Among Adolescents in Europe and North America 1994–2022
Source: Int J Public Health. 2025 Feb 24;70:1607654. doi: 10.3389/ijph.2025.1607654 (PMC11891013; doi:10.3389/ijph.2025.1607654)

SUPPLEMENTAL TABLES AND GRAPHS

Supplemental Figure 2a. Absolute change in the prevalence of fighting from 2002 to 2022 in 19 countries, by gender and age group (Health Behaviour in School-aged Children study, 2002–2022 for 19 countries).

Supplemental Figure 2b. Absolute change in the prevalence of bullying perpetration from 1994 to 2022 in 19 countries, by gender and age group (Health Behaviour in School-aged Children study, 1994–2022 for 19 countries).

Supplemental Figure 2c. Absolute change in the prevalence of bullying victimization from 1994 to 2022 in 19 countries, by gender and age group (Health Behaviour in School-aged Children study, 1994–2022 for 19 countries).

Supplemental Figure 3a. Absolute change in the prevalence of cyberbullying perpetration from 2018 to 2022 in 18 countries, by gender and age group (Health Behaviour in School-aged Children study, 2018–2022 for 18 countries).

Supplemental Figure 3a. Absolute change in the prevalence of cyberbullying victimization from 2018 to 2022 in 18 countries, by gender and age group (Health Behaviour in School-aged Children study, 2018–2022 for 18 countries).

Supplemental Figure 2a. Absolute change in the prevalence of fighting from 2002 to 2022 in 19 countries, by gender and age group

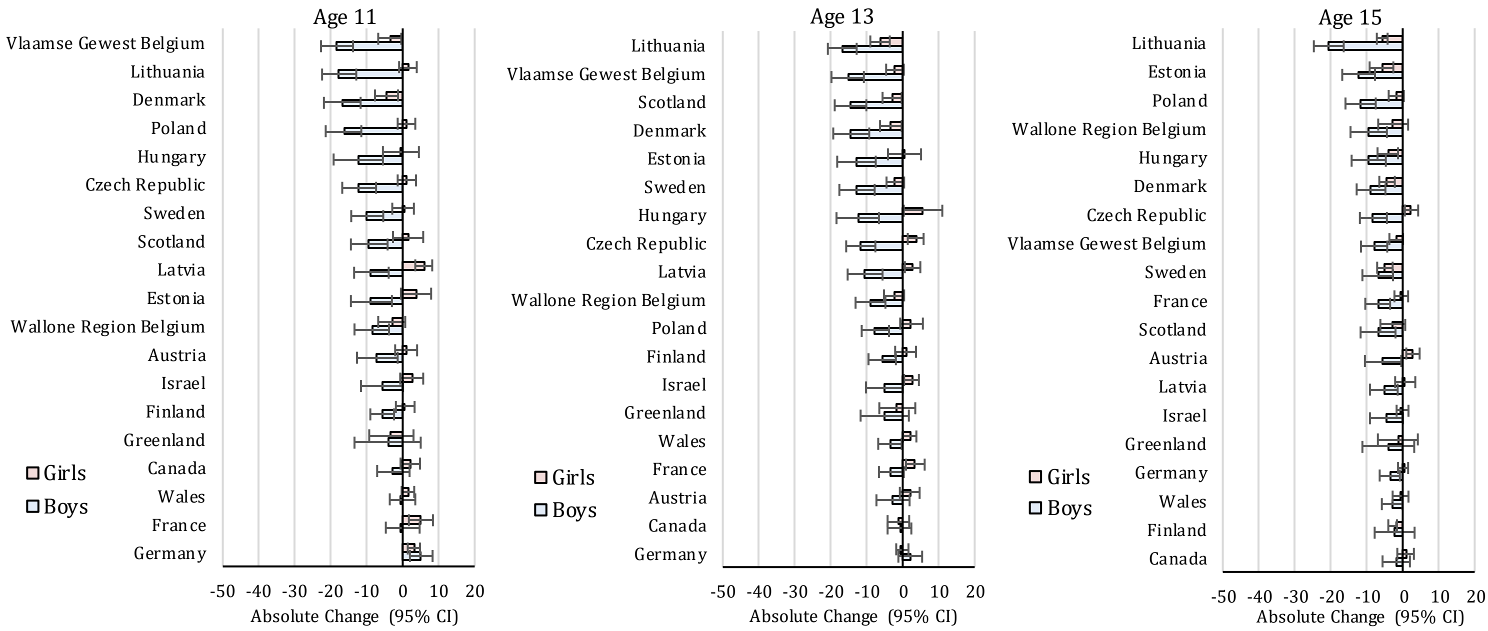

Supplemental Figure 2b. Absolute change in the prevalence of bullying perpetration from 1994 to 2022 in 19 countries, by gender and age group

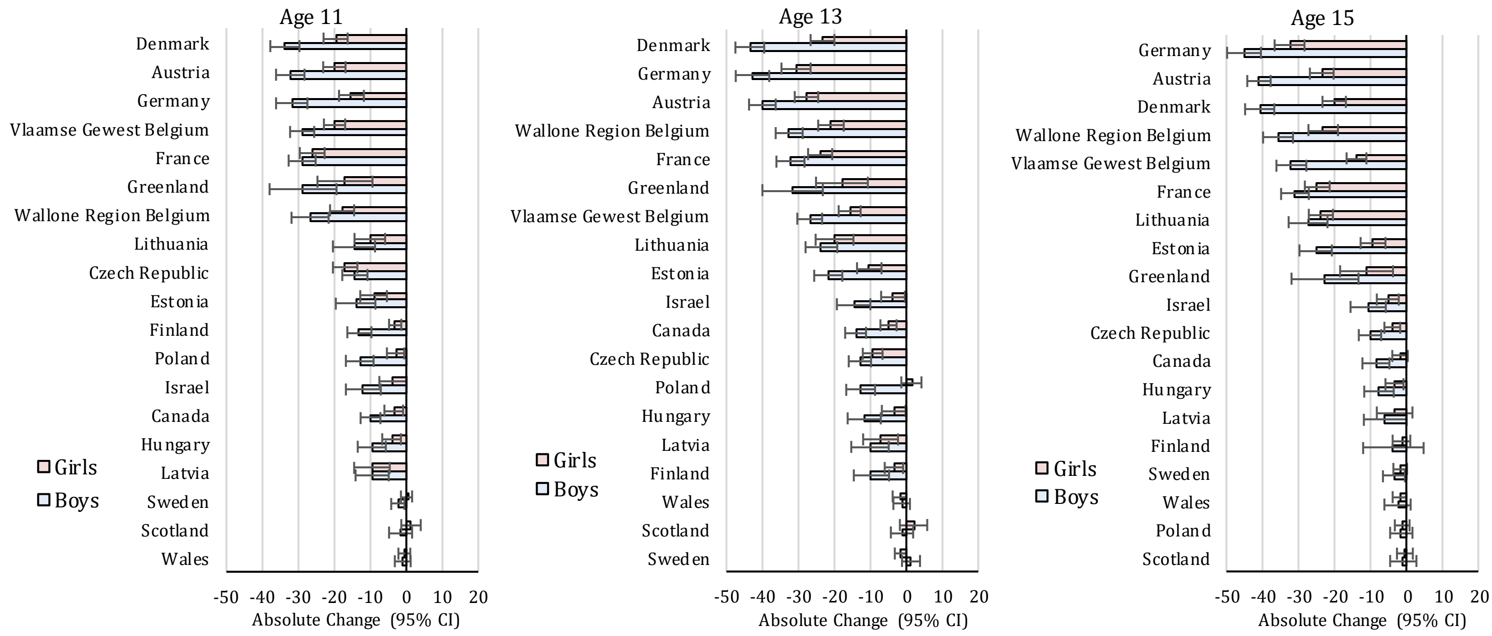

Supplemental Figure 2c. Absolute change in the prevalence of bullying victimization from 1994 to 2022 in 19 countries, by gender and age group

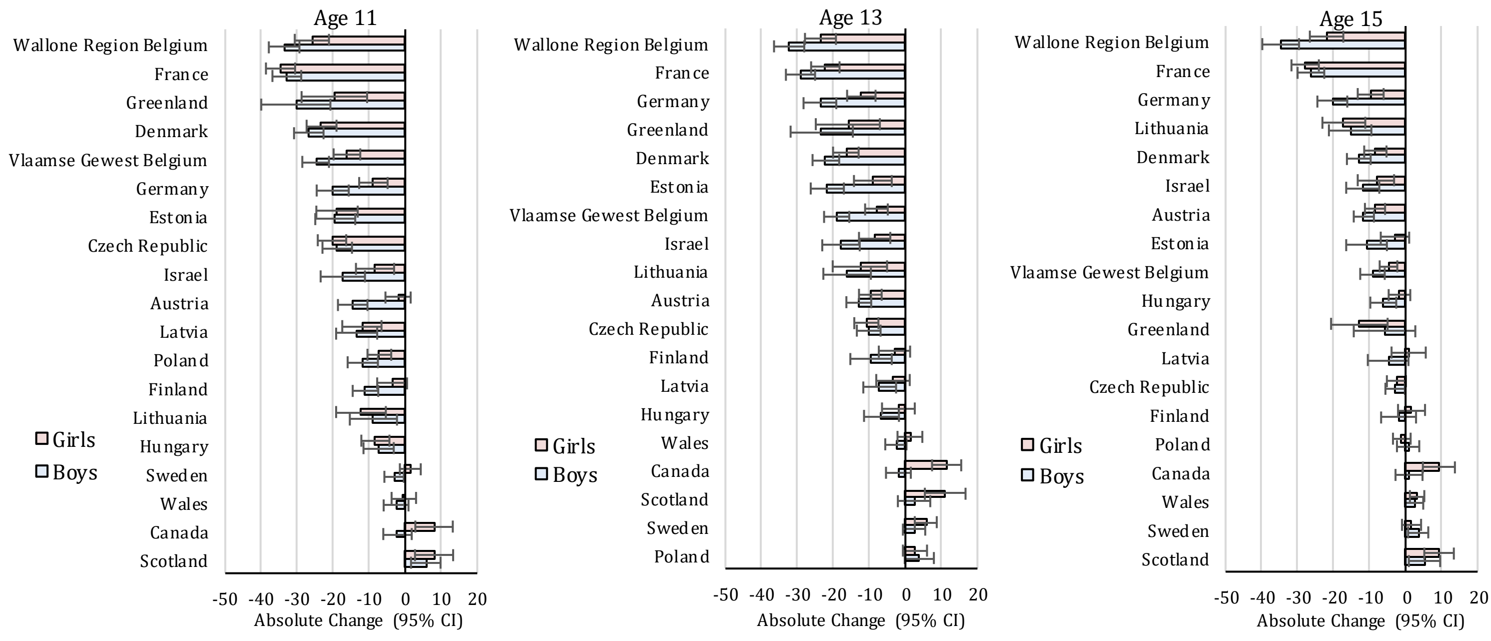

Supplemental Figure 3a. Absolute change in the prevalence of cyberbullying perpetration from 2018 to 2022 in 18 countries, by gender and age group

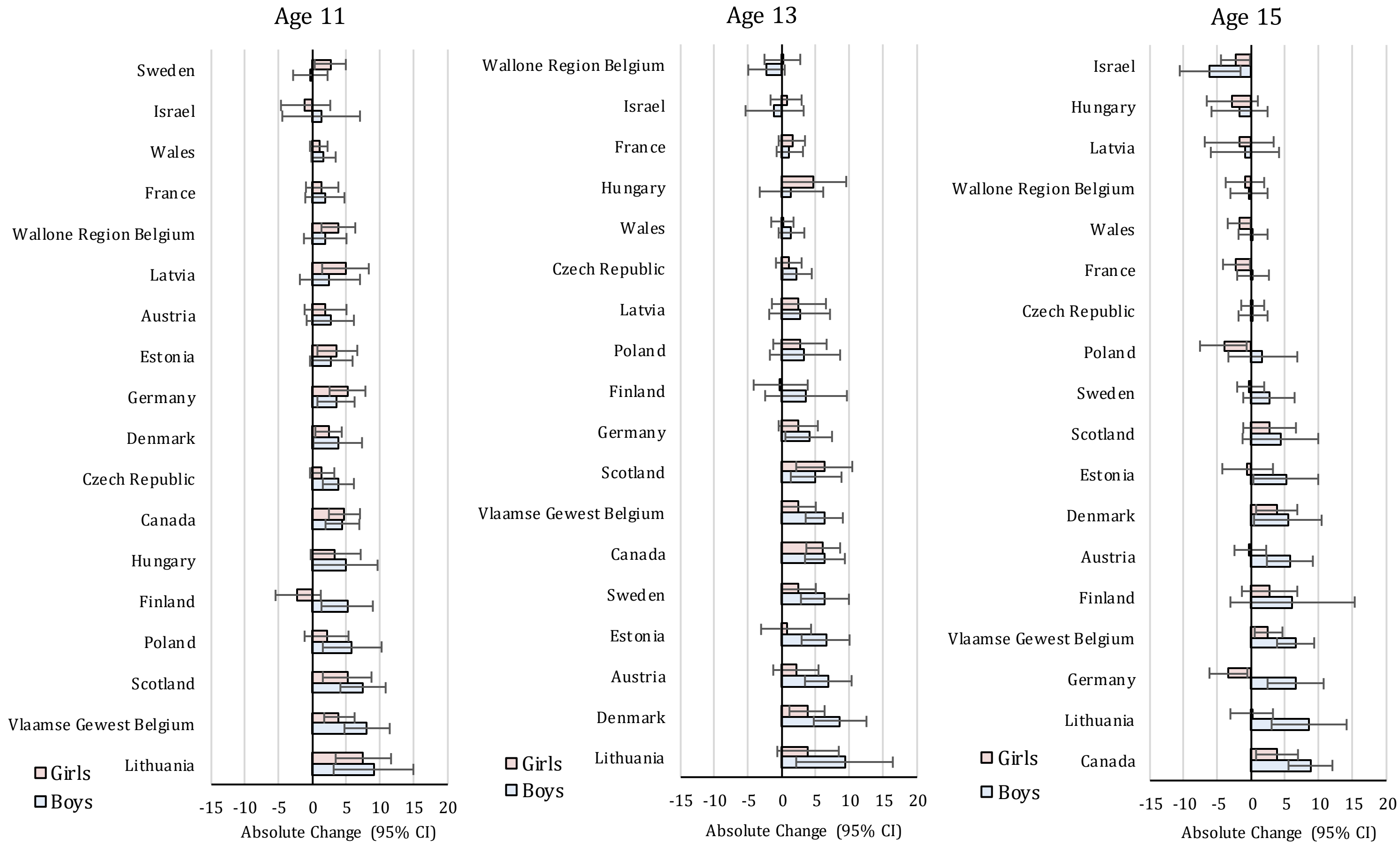

Supplemental Figure 3b. Absolute change in the prevalence of cyberbullying victimization from 2018 to 2022 in 18 countries, by gender and age group

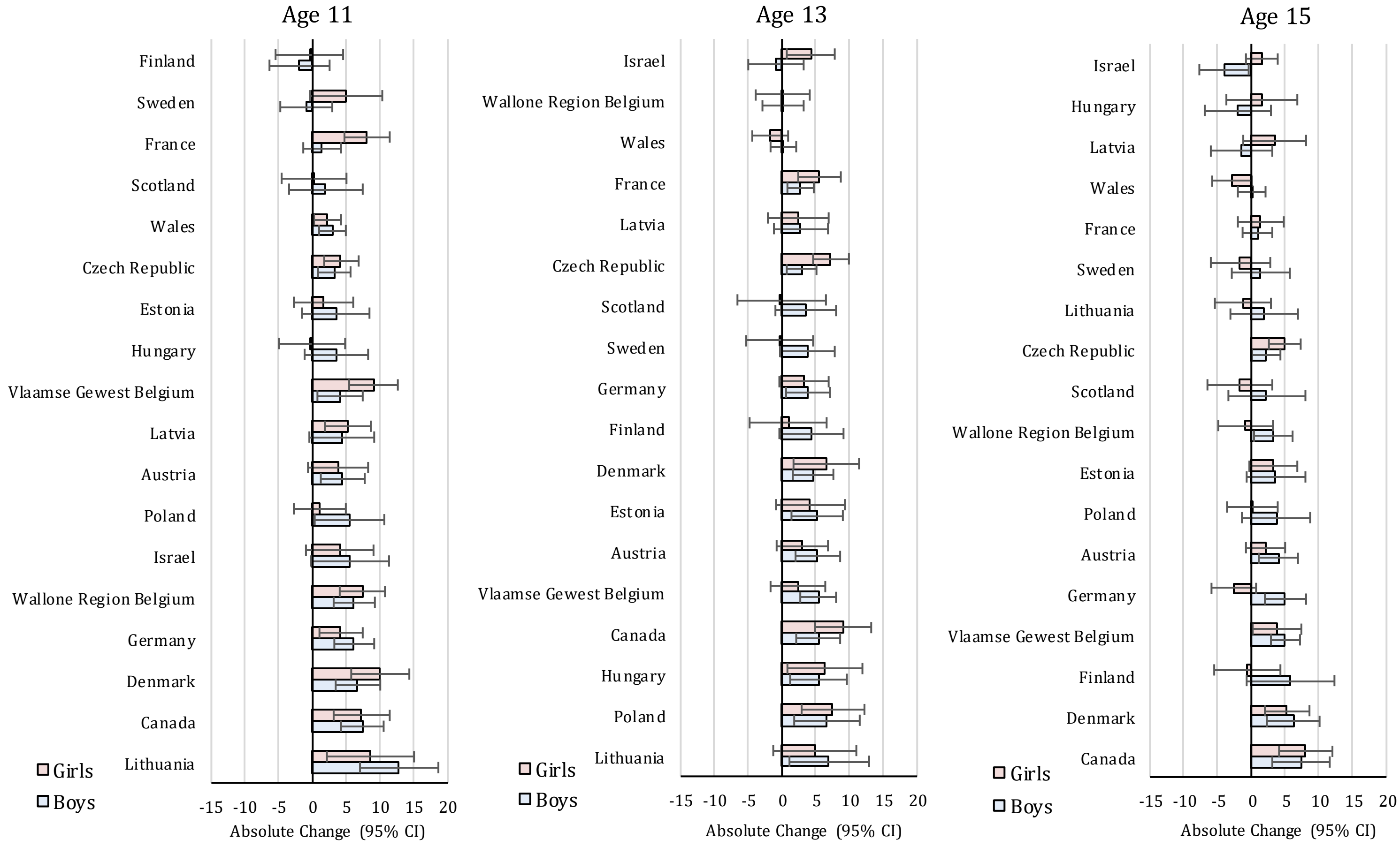

Supplement: Supplementary file 1 [file DataSheet1.pdf]
